# Traumatic shoulder fracture-dislocation in a 7-year-old child: a case report

**DOI:** 10.1186/1752-1947-7-156

**Published:** 2013-06-20

**Authors:** Mustafa Isik, Mehmet Subasi, Oguz Cebesoy, Irfan Koca, Ugur Pamukcu

**Affiliations:** 1 Department of Orthopaedics and Traumatology, University of Gaziantep Faculty of Medicine, Gaziantep, Turkey; 2 Department of Physical Medicine and Rehabilitation, University of Gaziantep Faculty of Medicine, Gaziantep, Turkey

## Abstract

**Introduction:**

In contrast to adults, traumatic glenohumeral dislocation is a rarely observed condition among children. In some cases, success in durable reduction with conservative methods, and achieving lasting treatment, may not be possible.

**Case presentation:**

In this study, the case of a 7-year-old Turkish girl with a Salter–Harris type II fracture and glenohumeral dislocation of the proximal humerus due to a fall from a height of 1.5 meters who underwent open reduction surgery is presented along with a review of the literature.

**Conclusion:**

Orthopedic surgeons should consider glenohumeral dislocation which is an extremely rare condition when they encounter proximal humerus fractures in pediatric trauma.

## Introduction

In contrast to adults, traumatic glenohumeral dislocation is a rarely observed condition among children. In some cases, success in durable reduction with conservative methods, and achieving lasting treatment, may not be possible [1]. In this study, the case of a 7-year-old patient with a Salter–Harris type II fracture and glenohumeral dislocation of the proximal humerus due to a fall from a height of 1.5 meters who underwent open reduction surgery is presented along with a review of the literature.

## Case presentation

**Figure 1:**
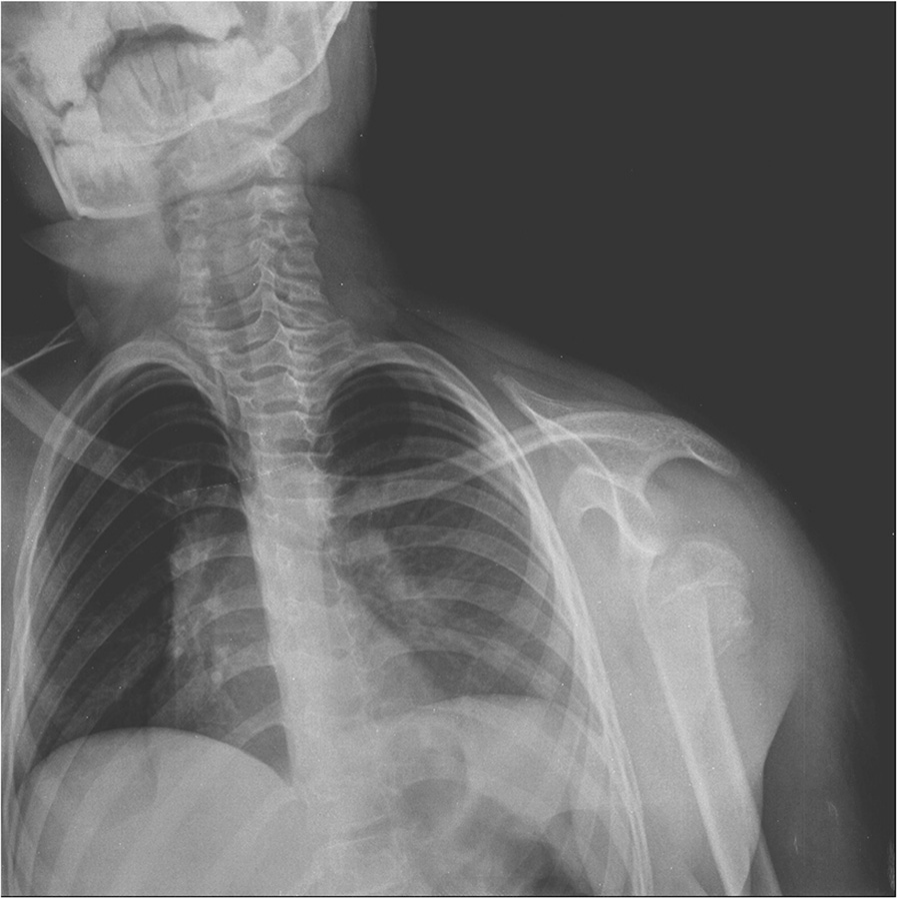
Radiologic image of complete glenohumeral dislocation and fracture line.

A 7-year-old Turkish girl with a history of a fall from a height of approximately 1.5 meters was seen on our ward. The patient had complaints of left shoulder pain and of being unable to move her shoulder. She preferred to stay seated in a slightly forward-leaning position, and was trying to support her elbow of the injured side with her other arm. A physical examination identified tenderness on her left shoulder, a slight swelling and limitation of movement. The patient was unable to perform movements with her left shoulder; her shoulder did not allow for passive movements as well. The patient’s neurovascular examination was entirely normal. On her radiography, a Salter–Harris type II epiphysiolysis at the proximal left humerus and a glenohumeral dislocation were identified (Figure 1). The epiphysis of the humerus appeared to be completely separated from the glenoid. After the preoperative preparations were complete, the patient was taken to the operating room.

**Figure 2:**
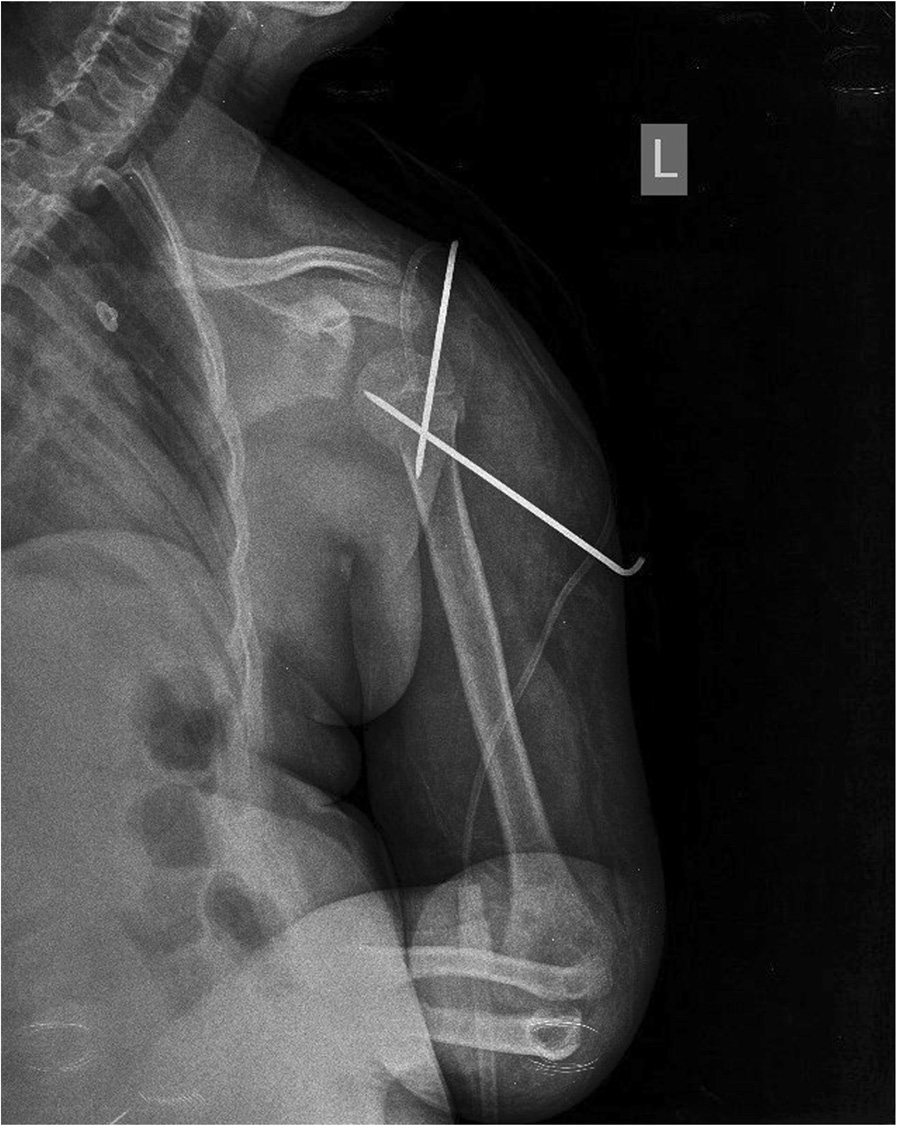
Radiologic image of shoulder postoperatively.

**Figure 3:**
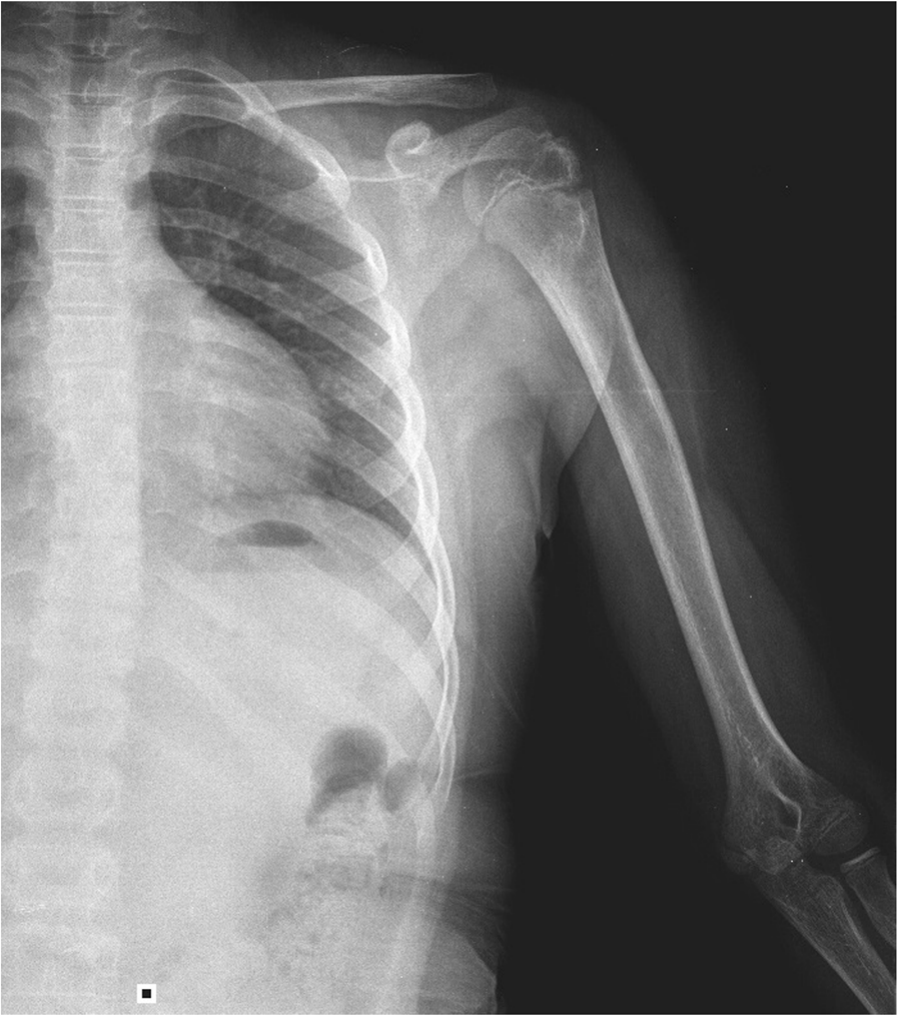
Radiologic image of shoulder after union achieved.

After fluoroscopy-guided closed reduction under general anesthesia was not successful, open surgery was performed instead. With a deltopectoral incision, the fracture line and the joint capsule were exposed. It was observed that the humerus head had moved beneath the glenoid. The fracture was first reduced, and glenohumeral reduction was then performed after fixation was ensured with two Kirschner wires (Figures 2 and 3). The patient was postoperatively followed-up for 4 weeks in Velpeau bandage. After union at the fracture was identified on the radiographs taken at the end of the postoperative 4th week, the Kirschner wires were removed under sedation, and the patient was commenced on an exercise program in order to increase the range of motion of her shoulder joint. After an exercise program of 2 weeks, the range of motion of the patient’s joint was almost fully restored. Only a limitation in abduction of 15 degrees was observed. In the postoperative 12th week, it was observed that the range of motion of her joint was at the same level as her healthy side. No redislocations were observed during the 6-month follow-up period.

## Discussion

Shoulder dislocations are very rarely observed in children. Rowe reported that only eight out of 500 patients with glenohumeral joint dislocation were under the age of 10 [1]. Proximal humerus fractures without glenohumeral dislocation are more frequent among children. Shoulder joint dislocations with proximal humerus physis injuries are even rarer [2,3]. Proximal humerus fractures are seen generally in early childhood and adolescence [4]. Closed reduction can be performed for treating Salter–Harris type I fractures. Open reduction is performed if closed reduction is unsuccessful. Salter–Harris type II injuries are observed more frequently [5]. However, Salter–Harris type III and type IV fractures as well epiphysis dislocations are very rarely observed [6]. The risk of recurrent dislocations due to inadequate immobilization and physiotherapy after reduction of the dislocated shoulder is higher [7]. Glenohumeral dislocations are generally encountered in case reports with accompanying proximal humerus fracture in the majority of the cases. Winmoon *et al.* reported the application of closed reduction and percutaneous Kirschner wire fixation for epiphysis slippage and glenohumeral dislocation in a 2-year-old child [8]. Do and Kellar reported the case of a 14-year-old patient with transitory inferior dislocation of the shoulder, whom they treated with percutaneous pinning [9]. The incidence of glenohumeral dislocation appears to increase with age. In a report published by Marans *et al.* on 21 shoulder dislocations they encountered over a period of 15 years, the average age for the patients was determined as 13 [10]. Concomitant glenohumeral dislocation and proximal humerus fractures are not expected in early childhood. Nugpok *et al.* reported the case of a 3-year-old child with a fracture-dislocation on the shoulder, which they treated in a similar fashion [11]. Based on the follow-up study they performed for 9 patients whose average age was 12 years and who had shoulder dislocations, Elbaum *et al.* reported an increased risk of recurrent dislocations among children with isolated glenohumeral dislocation [12]. Our patient was 7-years old, and had an inferior glenohumeral dislocation and a proximal fracture of the humerus. This injury, which is very rarely reported in the literature, was treated with open reduction and Kirschner wire fixation. There were no redislocations during the follow-up period after treatment.

## Conclusions

Shoulder joint dislocations with or without proximal humerus fracture are commonly encountered injuries in adults. By contrast, shoulder joint dislocations or fracture-dislocations are an unexpected type of injury in childhood. There are only a limited number of studies in the literature on this subject. Orthopedic surgeons should consider glenohumeral dislocations when they encounter proximal humerus fractures in children, especially after trauma.

## Consent

Written informed consent was obtained from the patient’s father for publication of this case report and accompanying images. A copy of the written consent is available for review by the Editor-in-Chief of this journal.

## Competing interests

The authors declare that they have no competing interests.

## Authors’ contributions

MI provided medical care and performed surgery on the patient. MS participated in the discussion. OC reviewed and edited the manuscript. IK managed the exercise program. UP collected related data with the patient. All authors read and approved the final manuscript.
